# Impact of smoking on the treatment cycle and health economics of acute exacerbations of chronic obstructive pulmonary disease

**DOI:** 10.18332/tid/219365

**Published:** 2026-06-05

**Authors:** Shanna Li, Li Yin, Mingze Lan, Gui Huang

**Affiliations:** 1 Department of Respiratory and Critical Care Medicine, Clinical Medical College and Affiliated Hospital, Chengdu University, Chengdu, China; 2 Department of General Medicine, Clinical Medical College and Affiliated Hospital, Chengdu University, Chengdu, China; 3 Department of Respiratory and Critical Care Medicine, Chengdu Pidu District People's Hospital, Chengdu, China

**Keywords:** chronic obstructive pulmonary disease acute exacerbation, smoking, treatment cycle, health economics

## Abstract

**INTRODUCTION:**

Acute exacerbations of chronic obstructive pulmonary disease (COPD) increase disease progression and mortality. Smoking influences treatment and health economics. This study examines how smoking status affects medical costs, hospital stays, and outcomes in acute COPD exacerbations.

**METHODS:**

We included 658 patients with acute COPD exacerbations, excluding those with asthma, allergic diseases, bronchiectasis, or tuberculosis. Participants were divided into never smokers (n=218) and smokers (n=440). Smokers were further classified as early smokers (age ≤24 years, n=70) or late smokers (age >24 years, n=370), and as current (n=164) or former smokers (n=276). We used Mann-Whitney U tests, chi-squared tests, and univariate logistic regression (p<0.05).

**RESULTS:**

Median hospital stay was 11 days (IQR: 8–16) for smokers versus 10 days (IQR: 7–15) for never smokers (p=0.179). Median daily cost was 1280.63 versus 1182.01 RMB (1000 Chinese Renminbi about US$151.4, mean exchange rate 2018) (p=0.217). However, total treatment costs were higher for smokers (median=13477.81 [IQR: 9096.81–22651.91] vs 12296.95 RMB [7954.42–20392.61]; Hodges-Lehmann difference: 1068.87 RMB, p=0.045). Medicare (government health insurance program in China) payments were higher (12389.82 [7528.24–20366.10] vs 10216.77 RMB [4691.43–17742.65]; difference: 2349.09 RMB, p<0.001), while out-of-pocket payments were lower for smokers (1005.00 [100.00–2500.00] vs 1527.03 RMB [960.00–4686.97]; difference: -600 RMB, p<0.001). Early smokers had higher costs, longer stays (difference: 1.0 day, p=0.019), higher daily costs (difference: 173.2 RMB, p=0.001), and greater out-of-pocket payments (difference: 328.5 RMB, p=0.008) than late smokers. Current smokers had higher Medicare and total expenditures than former smokers (p=0.011 and p=0.010, respectively).

**CONCLUSIONS:**

Smoking, especially early smoking, significantly increases the economic burden of acute COPD exacerbations without affecting hospital stay duration.

## INTRODUCTION

Chronic obstructive pulmonary disease (COPD) is a chronic inflammatory lung disease characterized by airflow limitation, with high global prevalence and mortality rates^1^. An acute exacerbation of COPD is a period of time when a patient's respiratory symptoms deteriorate acutely beyond their daily variability and require additional treatment^2,3^. During this period, patients' condition worsens and healthcare needs increase, leading to a significant rise in healthcare spending. The Global Chronic Obstructive Lung Disease (GOLD) 2023 report highlights widespread underdiagnosis/misdiagnosis of COPD, which makes it difficult for patients to receive timely and appropriate treatment, leading to a significant increase in the frequency of healthcare resource use and a significant increase in healthcare costs^4^. It is important to note that AECOPD is actually the major expenditure component of healthcare costs for patients with COPD. Domestic studies in China have shown that the average cost per hospitalization for AECOPD hospitalized patients is as high as 11598 RMB (1000 Chinese Renminbi about US$151.4, mean exchange rate 2018) per hospitalization^5^. Smoking is a major risk factor for COPD, and about 80–90% of COPD deaths are related to smoking^6,7^. Chronic smoking can aggravate COPD by causing airway inflammation and alveolar destruction^8-10^. In addition, smoking is also associated with an increased risk of acute exacerbation of COPD, leading to increased hospitalization and higher medical expenses^11^. The acute exacerbation of COPD has a huge impact on the quality of life of the patients and also imposes a heavy economic burden on the family and society.

In China, the high prevalence of smoking, especially among men, has made COPD one of the major public health problems in China^12^. In recent years, despite the success of tobacco control policies and health education, the impact of smoking on COPD remains a serious challenge. Therefore, it is important to study the impact of smoking on the treatment cycle and health economics of patients with acute exacerbation of COPD to develop effective tobacco control measures and health policies.

The aim of this study was to analyze the effects of smoking status and age of smoking onset on the treatment cycle and health economics of patients with acute exacerbation of COPD, and to investigate the effects of smoking on the medical expenses, hospitalization days, and treatment outcomes of patients with acute exacerbation of COPD, so as to provide scientific basis for the tobacco control policy, and to provide reference for the clinical management of patients with acute exacerbation of COPD.

## METHODS

### Research design

The present study employed a retrospective design and encompassed all consecutive patients with acute exacerbation of chronic obstructive pulmonary disease (AECOPD) in conjunction with pneumonia who were admitted to the Clinical Medical College and Affiliated Hospital, Chengdu University, between December 2017 and December 2018.

COPD was diagnosed based on the Global Initiative for Chronic Obstructive Lung Disease (GOLD) criteria, defined as a post-bronchodilator forced expiratory volume in 1 second (FEV_1_) to forced vital capacity (FVC) ratio <0.70^4^. Acute exacerbation of COPD was defined as an acute worsening of respiratory symptoms (increased dyspnea, cough, and/or sputum production) beyond normal day-to-day variation, requiring additional treatment^2,3^.

Among the inclusion criteria, we excluded those patients with other respiratory diseases, such as bronchial asthma, allergic diseases, bronchiectasis, or tuberculosis, in combination. Based on the patients' smoking history, we categorized the study population into two groups: never smokers and smokers, and further subdivided the smokers into early smokers (age ≤24 years) and late smokers (age >24 years) based on the age of smoking initiation The cutoff of 24 years was selected based on evidence that alveolar development and lung maturation is typically complete by early adulthood, approximately at the age of 24 years. Smoking during this critical developmental window may cause irreversible structural damage to the developing lung, leading to more severe COPD phenotypes and worse clinical outcomes compared to smoking initiation after lung development is complete^6^. In addition, we also categorized smokers into former smokers (who had successfully quit smoking) and current smokers (who had not quit and continued to smoke) based on their smoking cessation status. Such grouping helped us to analyze in depth the impact of different smoking behaviors on the healthcare economic burden of AECOPD patients.

### Data collection

In this study, we collected basic information and clinical data. The information we collected included basic data on patients' gender, age, and disease history. For smoking-related indicators, we recorded key parameters such as patients' age of initiation of smoking, smoking age, and smoking index. In addition, we included the results of routine blood tests at the time of admission, including white blood cell count (WBC), neutrophil count (NEUT), eosinophil count (EOS), basophil count (BASO), and platelet count (PLT), as well as biochemical markers such as blood urea nitrogen (BUN), procalcitoninogen (PCT), and alanine aminotransferase (ALT), systolic blood pressure (SBP) and diastolic blood pressure (DBP). These parameters were selected as potential confounders or mediators that could influence treatment intensity and healthcare costs. Inflammatory markers (WBC, NEUT, EOS, BASO, PLT) reflect the severity of COPD exacerbation and infection; BUN and ALT indicate renal and hepatic function which may affect medication dosing and complications; PCT is a specific marker for bacterial infection that guides antibiotic use; and blood pressure parameters reflect cardiovascular comorbidity burden – all factors that may independently affect hospital resource utilization and costs. We also considered the comorbidities of the patients.

To fully assess the impact of smoking on the financial burden of healthcare, we recorded the patients' total costs, out-of-pocket and medically paid expenses, and the average number of days of hospitalization which serves as an important indicator of disease management. The cost analysis was conducted from a healthcare payer perspective, incorporating both government insurance (Medicare) payments and patient out-of-pocket expenditures. Only direct medical costs related to the index hospitalization were collected from the hospital billing system, including medications, diagnostic tests, therapeutic procedures, and bed charges. Indirect costs (e.g. lost productivity, transportation, caregiver burden) were not assessed. All costs are reported in RMB as recorded in the original billing data (1000 Chinese Renminbi about US$151.4, mean exchange rate 2018).

Missing data occurred in <2% of records. Smoking history was complete for all included patients; 12 patients with incomplete smoking data were excluded. Laboratory values missing in <1% of cases were handled via complete case analysis. Hospital cost and length-of-stay data were 100% complete as extracted from electronic billing records.

### Statistical analysis

Continuous variables were expressed as mean and standard deviation (SD) and analyzed by t-test. Nonparametric data were expressed as median with interquartile range (IQR) and analyzed using the Mann-Whitney U test. For categorical variables frequencies and percentages were used, and the chi-squared test was used for univariate analysis. All tests were two-tailed. Differences were expressed as statistically significant at p<0.05. SPSS 26.0 (IBM, New York, USA) software was used for all statistical analyses.

For cost comparisons, due to highly skewed distributions, we used nonparametric Mann-Whitney U tests with the Hodges-Lehmann estimator for median differences. Univariate logistic regression was used to calculate odds ratios (ORs) for insurance payment type comparisons. While generalized linear models with a gamma distribution would be appropriate for skewed cost data, we utilized nonparametric methods given our sample size and distribution characteristics.

As this was a retrospective study of consecutive patients over a fixed time period, no *a priori* power calculation was performed. *Post hoc* power analysis indicates we had >80% statistical power to detect the observed cost differences at α=0.05.

## RESULTS

### Baseline patient characteristics

The proportion of males, history of COPD disease, and FEV_1_/FVC ratio of patients in the smoking group were significantly different from those in the never smoking group (p<0.05). The median age of patients in the smoking group was 76 years (IQR: 68.00–83.00), which was significantly less than that of 80 years (IQR: 73.25–84.75) in the never smoking group (p<0.001), and the proportion of elderly in the smoking group was lower than that in the never smoking group (81.59% vs 95.41%). The lung function damage index (FEV_1_/FVC %) of patients in the smoking group was 47.90%, which was significantly lower than that of the never smoking group (58.14%) (p<0.001). In addition, there was a statistically significant difference in the number of comorbidities between the smoking and never smoking groups (p=0.010). Blood urea nitrogen (BUN) (5.53 mmol/L [IQR: 4.19–7.20]) was significantly higher in the smoking group than in the never smoking group (4.99 mmol/L [3.70–6.90]) (p=0.014). Systolic blood pressure was significantly lower in the smoking group than in the never smoking group (p=0.036), and the difference in diastolic blood pressure between the two groups was not statistically significant. In addition, there were no statistically significant differences in white blood cell count (WBC), neutrophils (NEUT), eosinophils (EOS), basophils (BASO), platelets (PLT), procalcitonin (PCT), and alanine aminotransferase (ALT) levels between the two groups (Table 1).

**Table 1:** Characteristics of participants, retrospective cohort study of smokers with acute COPD exacerbation at the Clinical Medical College and Affiliated Hospital, Chengdu University, China, December 2017–December 2018 (N=658)

*Characteristics*	*Never smoking group* *(N=218)* *n (%)*	*Smoking group* *(N=440)* *n (%)*	*χ²/Z*	*p*
**Gender**			276.34	<0.001
Male	82 (37.61)	422 (95.91)		
Female	136 (62.39)	18 (4.09)		
**Age** (years), median (IQR)	80.00 (73.25–84.75)	76.00 (68.00–83.00)	-4.65	<0.001***
≥65	208 (95.41)	359 (81.59)	23.37	<0.001***
<65	10 (4.59)	81 (18.41)		
**COPD disease history** (years), median (IQR)	10.00 (3.50–20.00)	10.00 (7.00–20.00)	-2.58	0.010**
FEV_1_/FVC (%), median (IQR)	58.14 (48.59–68.07)	47.90 (39.55–60.65)	-5.03	<0.001***
**Number of comorbidities**			11.45	0.010**
0	39 (17.89)	111 (25.23)		
1	103 (47.25)	178 (40.45)		
2	45 (20.64)	114 (25.91)		
3–5	31 (14.22)	37 (8.41)		
**GOLD**			33.42	<0.001***
1	31 (14.22)	29 (6.59)		
2	60 (27.52)	98 (22.27)		
3	36 (16.51)	103 (23.41)		
4	18 (8.26)	91 (20.68)		
Unknow	73 (33.49)	119 (27.05)		
**Measurements**	**Median (IQR)**	**Median (IQR)**		
WBC (×10^9^/L)	7.15 (5.54–9.91)	7.32 (5.64–10.21)	-0.95	0.344
NEUT (×10^9^/L)	5.12 (3.70–7.80)	5.31 (3.61–8.32)	-0.41	0.680
EOS (×10^9^/L)	0.08 (0.02–0.18)	0.11 (0.03–0.21)	-1.74	0.083
BASO (×10^9^/L)	0.03 (0.02–0.04)	0.03 (0.02–0.04)	-0.37	0.713
PLT (×10^9^/L)	168.00 (133.00–229.00)	167.00 (130.00–212.50)	-0.58	0.564
BUN (mmol/L)	4.99 (3.70–6.95)	5.54 (4.20–7.21)	-2.53	0.011
PCT (mg/L)	0.05 (0.02–0.20)	0.05 (0.02–0.23)	-0.42	0.675
ALT (U/L)	16.00 (12.00–23.00)	17.00 (12.00–26.00)	-0.82	0.411
SBP (mmHg)	127.00 (120.00–138.75)	125.00 (120.00–134.25)	-2.10	0.036
DBP (mmHg)	78.00 (71.00–82.00)	80.00 (72.00–81.00)	-1.16	0.245

FEV_1_: forced expiratory volume in 1 second. FVC: forced vital capacity. WBC: white blood cell count. NEUT: neutrophil count. EOS: eosinophil count. BASO: basophil count. PLT: platelet count. BUN: blood urea nitrogen. PCT: procalcitonin. ALT: alanine aminotransferase. SBP: systolic blood pressure. DBP: diastolic blood pressure. IQR: interquartile range. *p<0.05.

** p<0.01.

*** p<0.001.

### Analysis of medical expenses

Median hospitalization days were 10 (IQR: 7–15) for the never smoking group and 11 (IQR: 8–16) for the smoking group. The difference did not reach statistical significance (Hodges–Lehmann estimator difference: 1.00 day, 95% CI: 0.00–1.00, p=0.179). The never smoking group had lower median daily costs (1182.01 RMB [990.60–1654.04]) than the smoking group (1280.63 RMB [1047.45–1628.58]), but the difference was not statistically significant (estimator difference: 44.18 RMB, 95% CI: -27.79–114.34, p=0.217).

The total cost per treatment episode was significantly lower in the never smoking group (12296.95 RMB [7954.42–20392.61]) than in the smoking group (13477.81 RMB [9096.81-22651.91]; estimator difference: 1068.87 RMB, 95% CI: -194.21–2348.71, p=0.045). Similarly, Medicare payments were lower in the never smoking group (10216.77 RMB [4691.43–17742.65]) than in the smoking group (12389.82 RMB [7528.24–20366.10]; estimator difference: 2349.09 RMB, 95% CI: 688.72–3929.11, p<0.001). However, out-of-pocket payments were higher in the never smoking group (1527.03 RMB [960.00–4686.97]) than in the smoking group (1005.00 RMB [100.00–2500.00]; estimator difference: -600 RMB, 95% CI: -926.00 – -257.62, p<0.001) (Table 2; and Supplementary file Figure 1).

**Table 2:** Differences in hospital days and treatment costs between smoking and never smoking groups, retrospective cohort study of smokers with acute COPD exacerbation at the Clinical Medical College and Affiliated Hospital, Chengdu University, China, December 2017–December 2018 (N=658)

	*Never smoking group (N=218)* *Median (IQR)*	*Smoking group (N=440)* *Median (IQR)*	*Actual* *difference*	*Hodges-Lehmann estimator* *(95% CI)*	*p*
**Days of hospitalization**	10.00 (7.00–15.00)	11.00 (8.00–16.00)	1.00	1.00 (0.00–1.00)	0.179
**Costs** (RMB)					
Average daily cost	1182.01 (990.60–1654.04)	1280.63 (1047.45–1628.58)	98.62	44.18 (-27.79–114.34)	0.217
Out-of-pocket payment	1527.03 (960.00–4686.97)	1005.00 (100.00–2500.00)	-522.03	-600 (-926.00 – -257.62)	<0.001***
Medical insurance payment	10216.77 (4691.43–17742.65)	12389.82 (7528.24–20366.10)	2173.05	2349.09 (688.72–3929.11)	0.003***
Total costs	12296.95 (7954.42–20392.61)	13477.81 (9096.81–22651.91)	1180.86	1068.87 (-194.21–2348.71)	0.045*

RMB: 1000 Chinese Renminbi about US$151.4, mean exchange rate in 2018. Mann-Whitney U test with Hodges-Lehmann estimator for median differences. IQR: interquartile range.

* p<0.05. **p<0.01.

*** p<0.001.

Further analysis of insurance payment distribution revealed that the out-of-pocket payment percentage for patients in the never smoking group was as high as 13.76%, compared to 7.95% in the smoking group, with a statistically significant difference between the groups (OR=0.542; 95% CI: 0.321–0.897, p=0.026) (Table 3).

**Table 3:** Distribution of medicare payments between smoking and never smoking groups, retrospective cohort study of smokers with acute COPD exacerbation at the Clinical Medical College and Affiliated Hospital, Chengdu University, China, December 2017–December 2018 (N=658)

*Payments*	*Never smoking* *group (N= 218)* *n (%)*	*Smoking group* *(N=440)* *n (%)*	*OR*	*95% CI*	*p*
Medicare insurance*	188 (86.24)	405 (92.05)	0.542	0.321–0.897	0.026*
**Out-of-pocket payment**	30 (13.76)	35 (7.95)			

* Government health insurance program in China. Univariate logistic regression was used to calculate odds ratios (ORs) with 95% confidence intervals (CIs).

### Impact of age of smoking initiation and cessation on health care spending

We categorized smokers who started during the alveolar developmental stage (age ≤24 years) as early smokers (n=70) and those who started after the age of 24 years as late smokers (n=370). Smoking duration was longer in early smokers (median=50.0 years [40.0–50.0]) than in late smokers (median=30.0 years [30.0–40.0], p<0.001). Total healthcare costs were significantly higher in the early smoking group (median=14987 RMB [9521–23796]) than in the late smoking group (median=10513 RMB [7928–13695]; estimator difference: 3519.6 RMB, 95% CI: 1729.8–5600.9, p<0.001). In addition, early smokers had longer hospital stays (estimator difference: 1.0 day, 95% CI: 0.0–3.0, p=0.019), higher daily costs (estimator difference: 173.2 RMB, 95% CI: 75.6–276.6, p=0.001), and greater out-of-pocket payments (estimator difference: 328.5 RMB, 95% CI: 0.0–742.6, p=0.008) compared to late smokers. Medicare payments were also significantly higher (estimator difference: 3618.9 RMB, 95% CI: 1418.3–5948.2, p<0.001) (Table 4).

**Table 4:** Impact of early versus late smoking initiation on healthcare expenditures, retrospective cohort study of smokers with acute COPD exacerbation at the Clinical Medical College and Affiliated Hospital, Chengdu University, China, December 2017–December 2018 (N=440)

	*Early smoking (N=70)* *Median (IQR)*	*Late smoking (N=370)* *Median (IQR)*	*Actual* *difference Median* *(IQR)*	*Hodges-Lehmann* *estimator (95% CI)*	*p*
**Smoking age** (years)	50.0 (40.0–50.0)	30.0 (30.0–40.0)	20.0	20.0 (10.0–20.0)	<0.001***
**Days of hospitalization**	11.00 (8.00–16.00)	9.00 (7.75–12.00)	2.0	1.0 (0.0–3.0)	0.019*
**Costs (RMB)**					
**Average daily cost**	1326 (1083–1671)	1189 (927.3–1424)	137.1	173.2 (75.6–276.6)	0.001***
**Out-of-pocket payment**	1087 (300.0–2625)	820 (0–1491)	267.0	328.5 (0.0–742.6)	0.008**
**Medical insurance payment**	13199 (8107–21594)	9665 (6587–13527)	3533.9	3618.9 (1418.3–5948.2)	<0.001***
**Total costs**	14987 (9521–23796)	10513 (7928–13695)	4474.1	3519.6 (1729.8–5600.9)	<0.001***

Early smoking defined as initiation at age ≤24 years (alveolar developmental stage); late smoking as initiation at age >24 years. Mann-Whitney U test with Hodges-Lehmann estimator for median differences. RMB: 1000 Chinese Renminbi about US$151.4, mean exchange rate in 2018. IQR: interquartile range. IQR: interquartile range.

* p<0.05.

** p<0.01.

*** p<0.001.

In a comparative analysis of healthcare economic burden between former smokers (n=276) and current smokers (n=164), we found that current smokers had significantly higher Medicare payments (13794.4 RMB [8201.2–21865.4] vs 10475.9 RMB [6850.1–18293.6]; estimator difference: 2292.3 RMB, 95% CI: 461.0–4059.9, p=0.011) and total expenditures (15322.4 RMB [9742.9–24055.8] vs 11492.5 RMB [8568.9–20068.8]; estimator difference: 2052.4 RMB, 95% CI: 463.1–3675.3, p=0.010) than former smokers, even though the median age of smoking initiation was similar (30.0 years for both). Although differences in hospitalization days, daily costs, and out-of-pocket payments did not reach statistical significance, this pattern suggests that smoking cessation may confer economic benefits, though the relationship requires further investigation (Table 5).

**Table 5:** Healthcare economic burden comparison between former and current smokers, retrospective cohort study of smokers with acute COPD exacerbation at the Clinical Medical College and Affiliated Hospital, Chengdu University, China, December 2017–December 2018 (N=440)

	*Current smoking (N=164)* *Median (IQR)*	*Former smoking (N=276)* *Median (IQR)*	*Actual* *difference*	*Hodges-Lehmann* *estimator (95% CI)*	*p*
**Smoking age** (years)	30.0 (30.0–50.0)	30.0 (30.0–40.0)	0	0.0 (0.0–10.0)	<0.001***
**Days of hospitalization**	11.0 (8.0–16.0)	10.0 (8.0–14.0)	1	1.0 (0.0–2.0)	0.213
**Costs** (RMB)					
Average daily cost	1309.9 (1084.6–1669.9)	1240.4 (984.4–1567.9)	69.6	73.0 (-9.6–154.9)	0.084
Out-of-pocket payment	1018.9 (200.0–2578.6)	1000.0 (0.0–2450.0)	18.9	0.0 (-36.5–262.0)	0.498
Medical insurance payment	13794.4 (8201.2–21865.4)	10475.9 (6850.1–18293.6)	3318.6	2292.3 (461.0–4059.9)	0.011*
Total costs	15322.4 (9742.9–24055.8)	11492.5 (8568.9–20068.8)	3829.9	2052.4 (463.1–3675.3)	0.010*

Former smokers (n=276) had successfully quit; current smokers (n=164) continued smoking at admission. Mann-Whitney U test with Hodges-Lehmann estimator for median differences. RMB: 1000 Chinese Renminbi about US$151.4, mean exchange rate in 2018. IQR: interquartile range.

* p<0.05. **p<0.01.

*** p<0.001.

## DISCUSSION

This study was meticulously designed with the overarching aim of delving deep into the impact of smoking behaviors, specifically focusing on the age of smoking initiation and smoking status (current vs former smokers), on the medical economic burden borne by patients with COPD during acute exacerbations. Through an exhaustive analysis of detailed data from 658 COPD patients, we found several significant and meaningful results that offer crucial insights into both the health and economic implications of smoking within this patient demographic.

Prior research has firmly established smoking as a predominant risk factor for COPD, with strong associations to disease progression, an escalated frequency of acute exacerbations, and a consequent upward spiral in medical costs^13,14^. Illustrative of this, a comprehensive systematic evaluation revealed that smokers faced twice the risk of experiencing COPD acute exacerbations compared to their never smoking counterparts^15^. Similarly, another study found that smokers endured 1.5 times the number of hospitalizations due to COPD acute exacerbations in contrast to never smokers^16^. Our current study not only corroborates these earlier findings but also refines the understanding by exploring the nuanced impacts of smoking.

When comparing smoking and never smoking groups, we observed notable economic disparities. While hospital length of stay and daily costs were comparable between groups, total treatment costs were significantly higher for smokers. This difference was particularly pronounced in Medicare payment costs, where smokers incurred substantially greater expenditures. Interestingly, the pattern for out-of-pocket payments differed, with never smokers bearing higher out-of-pocket costs than smokers. These findings illustrate the disproportionate economic strain that smoking imposes on the healthcare financing framework during COPD acute exacerbations. From a practical clinical and policy-making vantage point, this knowledge equips healthcare providers with the impetus to offer more targeted financial guidance to patients, underlining the potential cost savings that can be reaped through smoking cessation. Policymakers, in turn, can utilize these data to design interventions such as subsidizing smoking cessation programs or augmenting insurance coverage for never smokers, all with the goal of alleviating the financial burden.

Further analysis of the smoking group by age of initiation revealed that early smokers bore a substantially greater healthcare burden than late smokers. Those who began smoking during the alveolar developmental stage (age ≤24 years) accumulated significantly higher total healthcare costs, with longer hospitalizations and greater daily expenditures. Early smokers also faced higher out-of-pocket costs compared to their later initiation counterparts. These results collectively point to the fact that early smoking inflicts more severe and enduring damage on the body, requiring greater healthcare resources and, hence, a heavier economic burden. This conclusion highlights the need to prevent early smoking initiation, especially among adolescents. Complementary research has consistently emphasized that early smoking not only heightens the risk of developing COPD but also accelerates disease progression and magnifies the economic burden^17,18^. For instance, longitudinal studies tracking adolescent smoking habits have revealed that those who start smoking in their teens are more likely to experience stunted lung function growth and face a substantially elevated risk of developing respiratory ailments later in life^19^.

The comparison between current and former smokers revealed that despite similar ages of smoking initiation, current smokers carried a disproportionately heavier economic burden. Current smokers required substantially higher Medicare payments and total healthcare expenditures compared to former smokers. While differences in hospitalization duration, daily costs, and out-of-pocket payments were not statistically significant, this pattern suggests that smoking cessation may confer economic benefits, though the relationship requires further investigation.

Beyond our findings, a substantial body of literature has revealed the prominence of COPD in the global economic burden of disease. According to the 2019 Global Burden of Disease Study, COPD accounted for 5.8% of global deaths and 3.2% of the global burden of disease (in terms of disability-adjusted life-years), ranking it as the third leading cause of death worldwide^20^. In 2010, the estimated annual cost of COPD in the United States was $49.9 billion, including $29.5 billion in direct medical expenses^21^. In China during the same period, the annual direct medical costs of COPD were $30.30 billion, direct non-medical costs were $1.36 billion, and hospitalization accounted for 56.7% of total costs^22^. Hospitalization costs were similarly exorbitant, with the average cost of hospitalization for patients with acute exacerbations of COPD amounting to 11598 RMB in China and £4400 in the UK. In the United States, the in-hospital mortality rate for COPD was approximately 4.3%, and the hospitalization cost per patient was $9545 in 2006^23^. A retrospective cohort nested case-control study in Spain further revealed that current smokers exhibited higher mean annual healthcare expenditures compared to former smokers (€3784 vs €2302)^24^.

However, there were relevant studies reporting higher healthcare utilization and costs associated with smoking cessation^25,26^. Individuals who had recently quit smoking were more likely to use more healthcare services within a year compared to current smokers^25–28^. These results may have been due to the fact that a patient's recently diagnosed major medical condition often leads to smoking cessation, which may attenuate the beneficial effects of smoking cessation in individuals who have recently quit when assessed cross-sectionally^29^. This phenomenon may be indicative of the patient's medical condition at the time of treatment initiation rather than the cessation of treatment itself^26^. Overall, the extant literature has indicated that short-term increases in healthcare costs may have been attributable to concomitant illnesses and quit attempts^30^. Nevertheless, smoking cessation remained a pivotal component of long-term healthcare cost reduction for smoking-related diseases, such as COPD, as it contributed to the delay and attenuation of the disease's onset and progression, thereby reducing the long-term consumption of healthcare resources and associated costs^31^. Indirect costs, such as loss of labor and home care resulting from COPD, accounted for approximately 30% of the total economic burden in the US^32^. According to the GOLD report, COPD was projected to be fifth in economic burden among diseases worldwide by 2030, with an anticipated escalation in its economic impact in the ensuing years^33^. These figures underscore the substantial impact of COPD on the global economy and underscore the pressing need to enhance COPD prevention and treatment strategies.

### Limitations

This study has limitations. Its retrospective, cross-sectional design precludes causal inference. Residual confounding likely exists as we did not adjust for socio-economic status, education, or detailed disease severity. Our healthcare payer perspective (Medicare + out-of-pocket) excluded indirect costs, and the single-center Chengdu setting limits generalizability. While we used nonparametric tests appropriate for skewed data, we did not employ advanced econometric models (e.g. GLM) that could adjust for multiple confounders. Recall bias for smoking initiation is possible, and we lacked data on smoking intensity and cessation duration. Finally, temporal trends during 2017–2018 were not assessed. Future prospective, multicenter studies with comprehensive cost perspectives and advanced modeling are needed.

## CONCLUSIONS

Our study presented a comprehensive understanding of the relationship between smoking and the economic burden borne by COPD patients during acute exacerbations. The results of this study underscore the substantial impact of smoking, especially early and current smoking, on the healthcare economic burden of COPD patients. The observed disparities between smoking and never smoking groups, early and late smokers, and current and former smokers all converged to highlight the crucial need for comprehensive tobacco control strategies. These strategies should focus on preventing early smoking initiation, particularly among vulnerable groups like adolescents, and promoting smoking cessation among current smokers. By implementing such strategies, we can potentially reduce the economic and health consequences of smoking-related COPD, improve patients' quality of life, and optimize the allocation of limited healthcare resources. Additionally, it is imperative to analyze how smoking interacts with other health risk factors to jointly exacerbate the healthcare economic burden of COPD patients, thereby providing strong evidence for tobacco control and cessation interventions and emphasizing the importance of early intervention.

## Supplementary Material



## Data Availability

The data supporting this research are available from the authors on reasonable request.
