# Supplementary material for: Impact of smoking on the treatment cycle and health economics of acute exacerbations of chronic obstructive pulmonary disease
Source: Tob Induc Dis. 2026 Jun 5;24:10.18332/tid/219365. doi: 10.18332/tid/219365 (PMC13241959; doi:10.18332/tid/219365)
Supplement: Supplementary file 1 [file TID-24-79-s1.pdf]

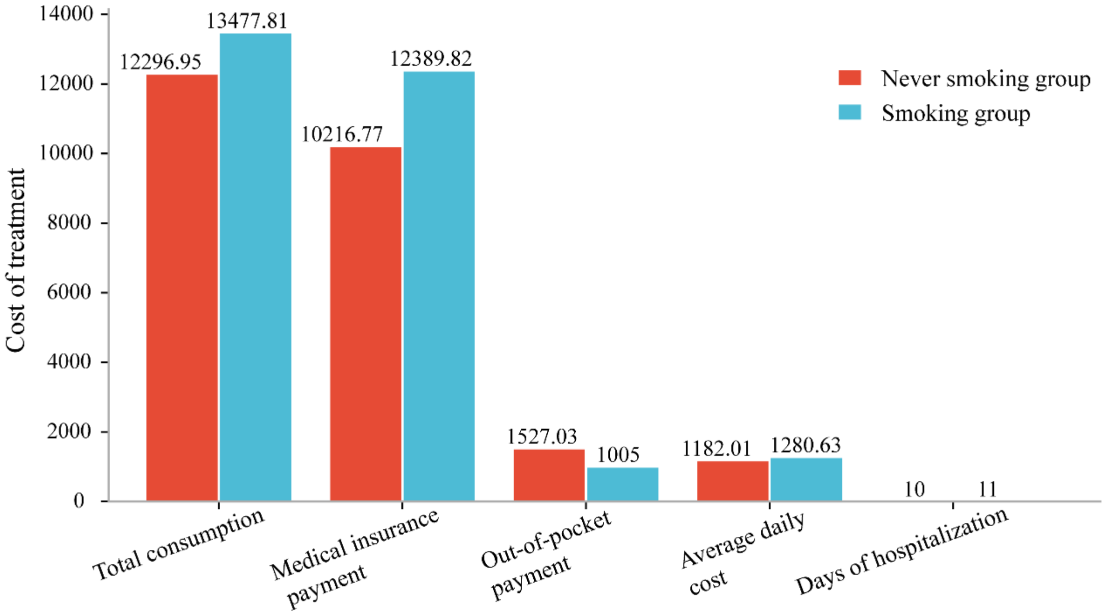

Supplementary Figure 1. Comparison of hospitalization days and treatment costs between smoking and never-smoking patients with acute COPD exacerbation. Retrospective cohort study, Chengdu, China, December 2017–December 2018 (N=658).

© **2026 Li S. et al.**
